# A technique to train new oculomotor behavior in patients with central macular scotomas during reading related tasks using scanning laser ophthalmoscopy: immediate functional benefits and gains retention

**DOI:** 10.1186/1471-2415-6-35

**Published:** 2006-11-23

**Authors:** Anouk Déruaz, Mira Goldschmidt, Andrew R Whatham, Christophe Mermoud, Erika N Lorincz, Armin Schnider, Avinoam B Safran

**Affiliations:** 1 Ophthalmology Clinic, Department of Clinical Neurosciences and Dermatology, Geneva University Hospitals, Switzerland; 2 Perception and Eye Movement Laboratory, Department of Neurology and Department of Clinical Research, University of Bern, Inselspital, Switzerland; 3 Division of Rehabilitation, Department of Clinical Neurosciences and Dermatology, Geneva University Hospital, Switzerland

## Abstract

**Background:**

Reading with a central scotoma involves the use of preferred retinal loci (PRLs) that enable both letter resolution and global viewing of word. Spontaneously developed PRLs however often privilege spatial resolution and, as a result, visual span is commonly limited by the position of the scotoma. In this study we designed and performed the pilot trial of a training procedure aimed at modifying oculomotor behavior in subjects with central field loss. We use an additional fixation point which, when combined with the initial PRL, allows the fulfillment of both letter resolution and global viewing of words.

**Methods:**

The training procedure comprises ten training sessions conducted with the scanning laser ophthalmoscope (SLO). Subjects have to read single letters and isolated words varying in length, by combining the use of their initial PRL with the one of an examiner's selected trained retinal locus (TRL). We enrolled five subjects to test for the feasibility of the training technique. They showed stable maculopathy and persisting major reading difficulties despite previous orthoptic rehabilitation. We evaluated ETDRS visual acuity, threshold character size for single letters and isolated words, accuracy for paragraphed text reading and reading strategies before, immediately after SLO training, and three months later.

**Results:**

Training the use of multiple PRLs in patients with central field loss is feasible and contributes to adapt oculomotor strategies during reading related tasks. Immediately after SLO training subjects used in combination with their initial PRL the examiner's selected TRL and other newly self-selected PRLs. Training gains were also reflected in ETDRS acuity, threshold character size for words of different lengths and in paragraphed text reading. Interestingly, subjects benefited variously from the training procedure and gains were retained differently as a function of word length.

**Conclusion:**

We designed a new procedure for training patients with central field loss using scanning laser ophthalmoscopy. Our initial results on the acquisition of newly self-selected PRLs and the development of new oculomotor behaviors suggest that the procedure aiming primarily at developing an examiner's selected TRL might have initiated a more global functional adaptation process.

## Background

Loss of reading ability is a leading complaint of subjects with central scotomas due to macular disorders, such as age-related macular degeneration (AMD) [e.g. [1,2]]. As fixation frequently shifts to a non-foveal location, an adaptation of the oculomotor system is necessary to search for, position and stabilize visual targets [3]. During such an adaptation, individuals develop one or several eccentric locations in the visual field that enable them to achieve fixation and reading [1,3-11]. These eccentric locations are referred to as preferred retinal loci (PRLs) [9]. Eccentric PRLs develop in approximately eighty-four percent of affected eyes [12]. The factors determining the development of a PRL at a precise location relative to the scotoma are still not well known. Previous studies reported that the PRL was located more frequently to the left of the scotoma (34% to 63% of the cases) [12,13]. Other studies showed that different PRLs could be used predictably according to various parameters, such as target size or background illumination [14-16]. In subjects with macular disorders, two factors strongly affect reading ability, namely global viewing of words related to the reading visual span, and character discrimination related to spatial resolution [17-19]. Global viewing of words refers to the ability to perceive the word in its entirety, to estimate its number of letters and to perform saccades to completely uncover it. Character discrimination refers to the ability to correctly read individual letters. Spontaneously selected PRLs do not consistently meet both requirements. In our previous investigations we found that multiple PRLs with complementary functional advantages could be used in a coordinated reading strategy [5,6,20] and we emphasized that the two essential capacities for reading could be allocated to different PRLs used in a combined fashion [5].

Whether and how individuals with central visual field loss could be trained in eccentric fixation is an important issue for the management of people suffering from age-related macular degeneration. A limited number of training procedures using the Scanning Laser Ophthalmoscope (SLO) to readapt fixation behavior during reading have been reported so far. Nilsson, Frennesson and Nilsson [21] investigated the influence of the SLO training structured in sessions scheduled to be one hour a week over two to seven weeks. In this study, subjects with age-related macular degeneration were trained to use a proposed TRL located above or below the lesion and to renounce to their spontaneously developed PRL. To our knowledge, the feasibility of a training process aiming at developing multiple PRLs has not yet been demonstrated.

Therefore the purpose of this investigation was to evaluate, in subjects with age-related macular degeneration, the use of the SLO to train eye movement strategies for reading, based on a combined use of two preferred retinal loci. The rationale for our selection of SLO training procedures was based on the observation that the use of more than one PRL in a structured reading strategy can meet the requirements for both global viewing and detailed discrimination [5-7,20]. Moreover, considering that intensive training over short periods of time is beneficial in neuro-rehabilitation [22-26], and that reorganization of the oculomotor system for eccentric reading can be achieved using an intensive training over a two-weeks period in experimental conditions [27], we designed our training protocol accordingly. We tested the feasibility of our procedure on subjects who reported an altered perception of words, despite conventional reading rehabilitation. Their main complaint was that they missed parts of words, thus expressing their lack of adapted global viewing abilities. We therefore attempted to train them to use in combination with their spontaneously developed PRL, an additional retinal locus, the trained retinal locus or TRL, fulfilling the needs for global viewing, i.e. allowing the perception of the entire word.

## Methods

The training procedure comprises three steps. Firstly, a baseline assessment of visual function and reading ability on isolated words is carried out. Secondly, the SLO training is conducted over ten sessions of one hour each. Finally, reading performance and reading strategies are evaluated both immediately after the completion of the SLO training process and three months later.

### General procedure

Subjects' evaluation includes visual acuity measurement, scotoma delineation, identification of spontaneously developed PRLs and characterization of oculomotor behavior during reading. Visual acuity is first determined for both eyes with ETDRS charts (Precision Vision Ltd, USA) (table 1). Based on the values obtained, the remaining evaluation and the training procedure are conducted in the eye with the best visual acuity. For the SLO experiments, subjects are comfortably seated forehead rested at 46 cm in front of the SLO screen, which provides images of 32 × 22 degrees of visual angle [7]. Stimuli are presented on the SLO screen at a luminance of 30 000 trolands (obtained using the conversion factor reported in Nygaard & Schuchard (2001) [28]). Experiments are conducted in a darkened room.

**Table 1:** Subject's clinical characteristics

**Subjects**	**Age** **(years)**	**Visual acuity (logMAR)**		**Selected eye**	**Contrast sensitivity in the selected eye (log unit)**
				
		**Right eye**	**Left eye**		
**MT**	81	1.1	1.43	RE	1.6
**SL**	76	1.46	2.2	RE	1.3
**LG**	80	1.24	1.5	RE	1.2
**AA**	78	1.12	1.02	LE	1.6
**GM**	79	1.5	1.36	LE	1.55

Visual acuity was measured with ETDRS charts. Selected eye: eye with the better visual acuity selected for training.

### Step I: initial evaluation

The scotoma is delineated using the microperimetry software of the scanning laser ophthalmoscope (SLO, Scotometry 3.0, Rodenstock, Germany). The testing method is based on the one described by Sunness *et al. *(1995) [29]. Briefly, the patient is instructed to fixate a cross. The investigator firstly chooses a retinal landmark on the SLO real-time image that serves as a reference point. Subsequently stimulus points are presented for 500 msec on sites of interest. The order of presentation is randomized. The patient has to press a button each time he sees a stimulus and the response is recorded on the computer. After the completion of the testing a final fundus image is grabbed, where the investigator specifies the position of the landmark. The data points are displayed on this image with different symbols for seen and unseen stimuli. Subsequently we present through the SLO to each subject a series of 32 words varying in length to enable the subjects to become familiar with the testing procedure.

Baseline parameters of the training procedure are evaluated for the threshold character size for single letters, isolated words and paragraphed text, for the accuracy of words recognition during paragraphed text reading and for the oculomotor strategies while reading isolated words and paragraphed text.

#### Evaluation of threshold character size for single letters and isolated words

Threshold character size for single letters and isolated words is assessed using three different lists of 32 items of 8 different character sizes (1.5 to 0.8 logMAR) that we project with the SLO in a random order onto the retina. Threshold character size is defined as the smallest size at which a correct verbal report of the isolated letter or word can be made at the first attempt. The results obtain for the three different lists are averaged. Isolated words are of three types: 3 and 4-letter words, 6 and 7-letter words and 9 and 10-letter words (table 2). Words are matched for frequencies (> 1/1000) and grammatical category between word-length using the Brulex database [30]. Matching words according to these two parameters makes impossible to use words of a single length in a group. Presenting words of different lengths in a random order is intended to restrict subjects' prediction of the word based on its number of letters. Each word is presented only once to each subject during the whole experiment.

**Table 2:** ETDRS acuity and threshold character sizes for single letters, isolated words and paragraphed text

	**ETDRS acuity (logMAR)**			**Threshold character size for words (logMAR)**														**Threshold character size for paragraphed text (logMAR)**		

**Patients**	**Before training**	**Immediately after training**	**3 months later**	**Before training**					**Immediately after SLO training**					**3 months later**				**Before training**	**Immediately after training**	**3 months later**
							
				**1**	**3 and 4**	**6 and 7**	**9 and 10**		**1**	**3 and 4**	**6 and 7**	**9 and 10**		**1**	**3 and 4**	**6 and 7**	**9 and 10**			

**MT**	1.1	0.96	0.9	1.0	1.1	1.4	1.4		0.9	1.3	1.2	1.3		0.9	1.2	1.4	1.5	1.4	1.3	1.5
**SL**	1.46	1.1	1.14	1.0	1.2	1.4	1.4		0.9	1.1	1.2	1.2		0.9	1.2	1.2	1.2	1.4	1.2	1.4
**LG**	1.24	1.2	1.12	1.1	1.2	1.5	1.4		0.8	1.0	1.2	1.0		0.8	0.8	1.1	1.2	1.4	1.2	1.2
**AA**	1.02	0.84	0.86	1.0	1.1	1.1	1.1		0.8	1.0	1.1	1.0		0.8	1.0	1.0	1.1	1.2	1.1	1.2
**GM**	1.36	1.2	1.1	1.15	1.5	> 1.5	> 1.5		0.8	1.1	1.2	1.1		0.9	1.2	1.2	1.3	1.5	1.4	1.5

1: 1 letter; 3 and 4: words of 3 and 4 letters; 6 and 7: words of 6 and 7 letters; 9 and 10: words of 9 and 10 letters. The mean value obtained for each patient and for each stimulus type is reported.

#### Evaluation of paragraphed text reading

Paragraphed text reading is evaluated using texts from elementary school handbooks (children between 8 and 12 years old) to reduce possible difficulties linked with lexicon and grammar contents. Threshold character size is evaluated using the same eight sizes previously used for isolated words and is defined as the smallest size at which the subject is able to read the text without complaining about character size. For each text size read, we evaluate reading accuracy by determining the percentage of words read correctly at first attempt for each character size. Presentation time of visual stimuli is not restricted.

#### Analyses of reading strategies

Analyses of reading strategies are conducted according to the method we previously described [5]. To summarize, the SLO is used in conjunction with a program developed in our laboratory for projecting letters, words or paragraphed texts directly onto the ocular fundus. Images of the ocular fundus and the superimposed words obtained during the reading task are recorded onto digital videotape, at a frequency of 25 frames/second. Another program developed in our laboratory allows extracting eye movements automatically by comparing the frame-by-frame shift of the image position [31]. Scotoma borders and some major retinal landmarks, such as the optic disc and blood vessels are outlined from the microperimetry results to produce a schematic image. Finally, the program reconstructs a "cartoon" of the sequence of the eye movements during reading (e.g. figures 3, 4, 5, 6, 7). From these cartoons, we analyse the oculomotor behavior and identify the PRLs employed. We defined a PRL as an area repeatedly and consistently used during reading isolated words and paragraphed text. This area is used by the patient to perform saccades and fixations while deciphering the presented word or segment of text.

**Figure 1:**
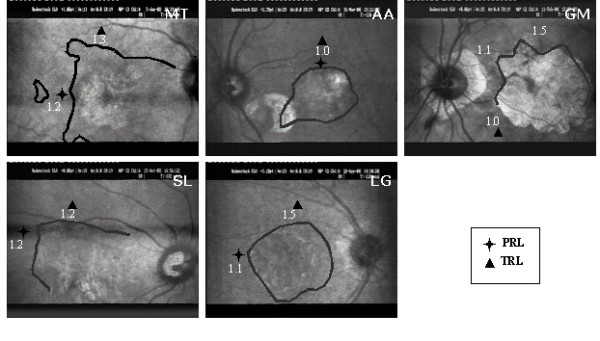
**Scotoma topography**. Scotoma borders were plotted using scotometry, with the brightest (Goldman III) stimulus. In subjects MT, SL and GM, parts of scotoma borders could not be delineated, as the scotoma was not always entirely visible within the SLO analysis area. Locations of the initial preferred retinal locus (PRL ) and the examiner's selected trained retinal locus (TRL ) are indicated, as well as the visual acuity measured at these locations with visumetry.

**Figure 2:**
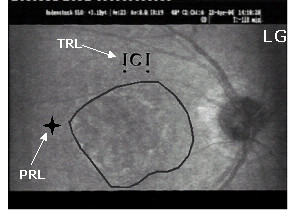
**Procedure for SLO training**. First, fixation was anchored using a cross at the initial preferred retinal locus (PRL) and words were projected onto the location of the examiner's selected trained retinal locus (TRL). Projected words and crosses were randomly presented at two different locations on the SLO screen to induce changes in eye position. The anchored cross was eventually removed and the subject was asked to repeatedly attempt to scrutinize the word alternating by himself between fixation locations.

**Figure 3:**
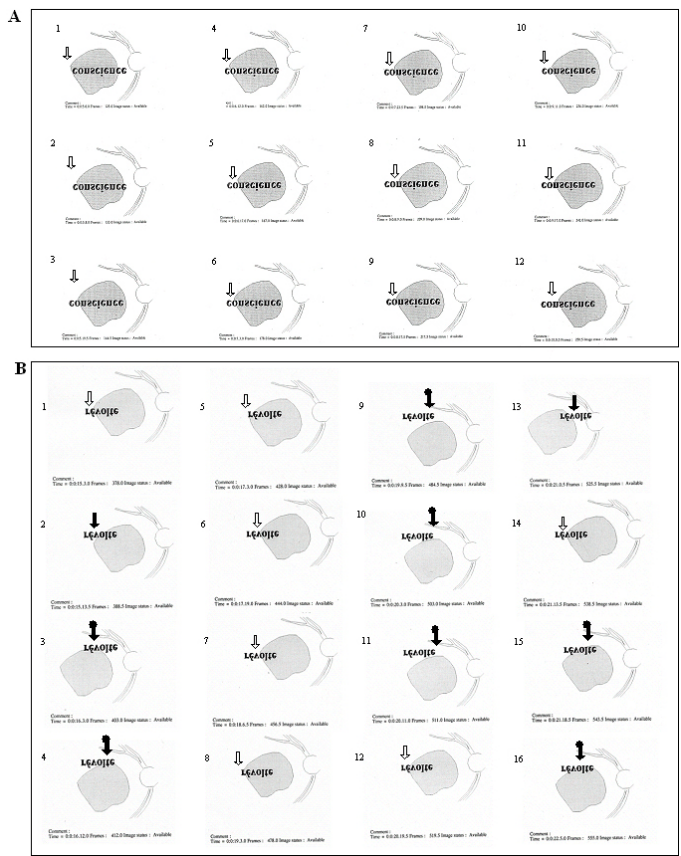
**The development of the combined use of initial PRL, examiner's selected TRL, and two new self-selected PRLs for reading isolated words – before and immediately after training**. The figure shows, as seen on SLO images, two uninterrupted sequences of eye movements performed while attempting to decipher an isolated word presented in 1.3 logMAR. Recordings were obtained in subject LG (A) before SLO training and (B) immediately after SLO training. In each reading sequence images are numbered in chronological order. Previous to SLO training, the inability to perform eye movements to reach the end of the word "conscience" prevented reading. The subject therefore only read "con". Immediately following SLO training, LG adapted his reading strategies and was able to read entirely the word "révolte". The arrows indicate the position of the retinal areas being presumably employed: initial PRL (), examiner's selected TRL () and newly self-selected PRLs (). The examiner's selected TRL was consistently used combined with the initial PRL and/or with newly self-selected PRLs. Please note that, on the SLO image, an upside down inversion of the image allows the word and the presented reading strategies to be viewed normally.

**Figure 4:**
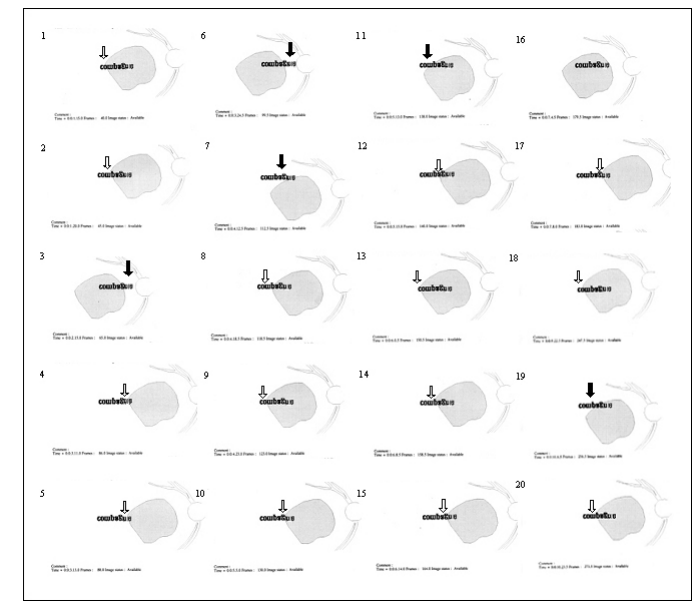
**The development of the combined use of initial PRL, examiner's selected TRL, and two new self-selected PRLs for reading isolated words – three months after training**. This figure shows an uninterrupted sequence of eye movements performed while attempting to decipher an isolated word presented in 1.3 logMAR, like in figure 3. Recordings were obtained in subject LG three months following the training procedure. Adaptation due to the training procedure was maintained allowing LG to read entirely the word "compagnie". The arrows indicate the position of the retinal areas being presumably employed: initial PRL () and newly self-selected PRLs (). The initial PRL was consistently used with newly self-selected PRLs. Please note that, on the SLO image, an upside down inversion of the image allows the word and the presented reading strategies to be viewed normally.

**Figure 5:**
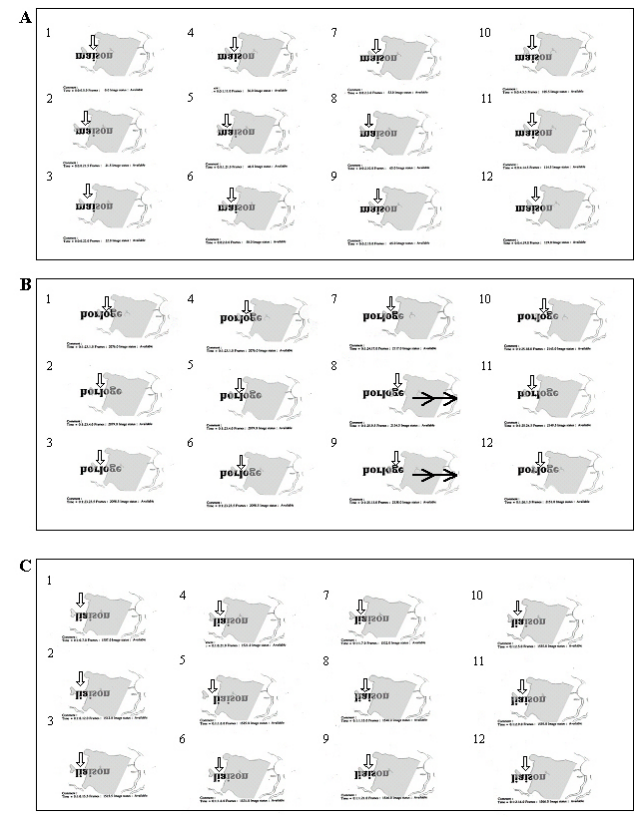
**The development, with SLO training, of a progressive uncovering strategy for reading isolated words**. The figure shows three uninterrupted sequences of eye movements while reading an isolated word presented in 1.5 logMAR as seen on SLO images. Recordings were obtained in subject MT (A) before SLO training, (B) immediately after SLO training and (C) three months later. In each reading sequence images are numbered in chronological order. Before training, the subject was unable to read entirely the word "maison". He read only "mais". Immediately following SLO training, he became able to read entirely the word "horloge", performing successive eye movements uncovering segment by segment the entire word with the same retinal area (). Three months following completion of the training process, however, the subject could not anymore read entirely the word "liaison". The vertical arrow indicates the retinal area presumably being employed, here exclusively the initial PRL (). MT was, in the study, the only subject of the experiment to lose both SLO training gains, and modification of the eye movement pattern. Note that, on the SLO image, an upside down inversion of positions allows the word and the presented reading strategies to be viewed normally.

**Figure 6:**
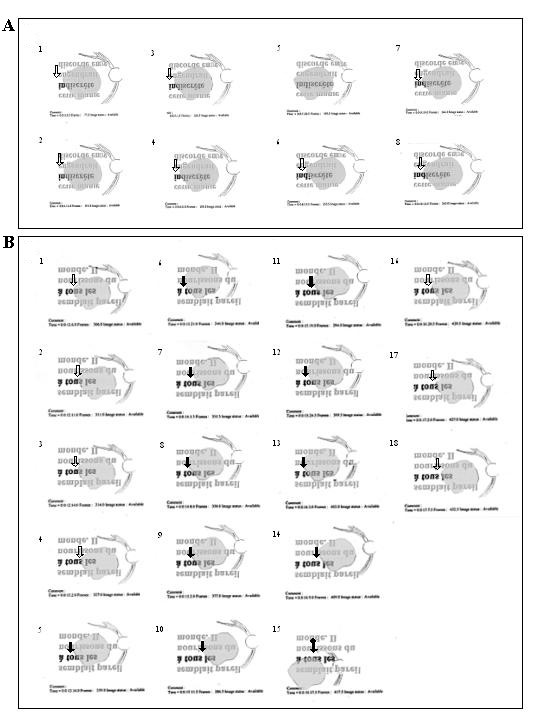
**The development, with SLO training, of combined use of the initial PRL and examiner's selected TRL for reading paragraphed text – before and immediately after training**. This figure shows, as seen on SLO images, two uninterrupted sequences of eye movements while reading lines of paragraphed text presented in 1.4 logMAR. Recordings were obtained for subject LG (A) before SLO training and (B) immediately after SLO training. Vertical arrows indicate the retinal area presumably being employed for fixation. The line being read is shown darker than the other lines. Note that previous to SLO training, the subject exhibited restricted eye movements. Immediately following SLO training, the subject used consistently the examiner's selected TRL () in combination with the initial PRL () and/or with a newly self-selected PRL (). Note that on the SLO image, the text begins at the lower left side of the figure and ends in the upper right corner. An upside down inversion of positions allows the text and the reading strategies to be viewed normally.

**Figure 7:**
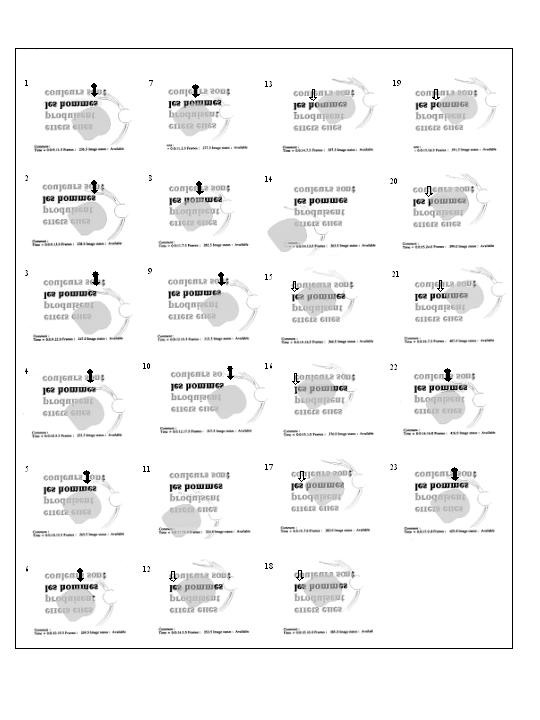
**The development, with SLO training, of combined use of the initial PRL and examiner's selected TRL for reading paragraphed text – three months after training**. This figure shows, an uninterrupted sequence of eye movements while reading lines of paragraphed text presented in 1.4 logMAR, like figure 6. Recordings were obtained for subject LG three months after training. Vertical arrows indicate the retinal area presumably being employed for fixation. Three months after training, the subject used consistently the examiner's selected TRL () in combination with the initial PRL () and/or with a newly self-selected PRL ().

### Step II: training procedure

As previously mentioned, reading with a central scotoma involves the use of PRLs that enable both letter resolution and global viewing of words. The spontaneously developed PRLs often privilege spatial resolution. We thus designed a procedure that intended to train the use of an additional area, the examiner's selected trained retinal locus (TRL), where the presented word could be perceived in its entirety. This area is generally situated either above or below the lesion on the SLO image. However, it is worth mentioning that our SLO approach is also valuable to train areas of high spatial resolution to optimize reading.

Among potentially valuable retinal locations, the area closest to the initial PRL is selected. Visual acuity is evaluated at the initial PRL and at the examiner's selected TRL (figure 1). This involves the presentation of a tumbling "E" in one of four orientations with the SLO using the visumetry program 3.0. Stimuli are presented in 0.1 logMAR steps. With this technique, visual acuity is defined as the smallest stimulus size at which a correct answer is obtained for three out of four presented stimuli.

The training process involves the projection of single letters and isolated words onto the retina using the SLO. To facilitate the control of eye position at the beginning of the procedure, fixation is anchored using a cross while words are projected onto the examiner's selected TRL (figure 2). Subjects are instructed to fixate the words by alternating between the initial PRL and the examiner's selected TRL. The PRL intends to fulfill one of both functions, i.e. letter discrimination or global viewing, and the TRL aims at fulfilling the remaining function. When applicable, subjects are trained to use the initial PRL for fine spatial discrimination and the examiner's selected TRL for global viewing, i.e. to estimate word length and to reach the end of the word. The anchored cross is eventually removed and the subject is asked to repeatedly attempt to scrutinize the word alternating by himself between these fixation locations. When the subject cannot voluntarily shift fixation onto the examiner's selected TRL, the training procedure is repeated from the previous stage using the fixation cross. Verbal feedback by the examiner assists in learning the use of the TRL.

In each training session the subject is asked to read a 32 single letters and isolated words' list, displaying a word-length distribution similar to that used in the pre-training evaluation, but including only four different character sizes. Character sizes are selected according to the threshold determined during the baseline testing, starting with two initially readable and two initially unreadable character sizes. Once the subject can read all words, the set of sizes is readjusted by removing the largest character size and including a smaller one. Our training process involves presenting the fixation cross and the associated word randomly at two different positions on the SLO screen to avoid subject's anticipation of the spatial location of the stimulus. Ten training sessions of one hour each are conducted at the same time of the day, with three to four sessions a week. We conduct the sessions at the same time each day to avoid variations of performances due to the circadian cycle. At the end of the SLO training, subjects are encouraged to apply the trained strategy at home. For this purpose, they are advised to use the reading device, which was prescribed by the low vision specialist during their previous rehabilitation training.

### Step III: post-training evaluations

To assess for possible functional changes, an evaluation is conducted both immediately after SLO training and three months later, and findings are compared to those collected before starting the training procedure. Our rationale for performing these two post-training evaluations is based on results obtained in normal subjects while learning to read using their eccentric visual field [27]. Those subjects were able to establish and develop an oculomotor strategy within two weeks but needed afterwards a period of at least one month to reach a plateau in performances [27] Assessment included ETDRS visual acuity and threshold character size for single letters and isolated words, reading accuracy for paragraphed text, as well as analysis of the oculomotor behavior and reading strategies. We finally ask the subjects to give his subjective impressions on the possible improvement linked to the training procedure.

## Results

To test for the feasibility of our training procedure, we conducted a pilot study involving 5 subjects.

### Subjects

Criteria used for inclusion of subject were the following: (1) presence of a bilateral AMD condition stable over the last year (table 1); (2) visual acuity ranging between 1.5 logMAR and 0.9 logMAR in the best eye; and (3) complaints of reading disturbances consisting essentially of gross difficulty to decipher long words. Six subjects were initially enrolled in the study (MT, SL, LG, AA, GM, ML). Subject ML, however, was subsequently discarded, as he showed severe signs of memory impairment three months following the training. His statement that he did not remember to have been trained in our laboratory emphatically demonstrated his memory loss. Ages ranged from 76 to 81 years (mean: 78.8 years ± 1.92 SD). In each subject a stable bilateral macular condition had been present in average for 2.3 years. Contrast sensitivity of subjects' best eye, as measured with the Pelli-Robson contrast sensitivity chart (Clement Clarke Intl, Haag-Streit, UK) was lower than the one measured in subjects without macular disorder of similar age [32]. These clinical features are shown in table 1. A regular low-vision rehabilitation procedure, including scanning and reading exercises similar to those described by Beaunoir *et al. *(2000) [33], had been conducted by a low vision specialist, for four to ten training sessions, extending over a period of more than three months. Despite rehabilitation, subjects presented reading difficulties during the SLO evaluation as for example reading only the first five letters of a ten letter-word, or guessing, often incorrectly the end of presented words. In general they were unable to uncover the end of words (table 3). Stability of the clinical condition and previous rehabilitation training allowed the use of each subject as his own control, as no spontaneous recovery was expected.

**Table 3:** Retinal areas used for reading and eye movements to completely uncover presented words

**Subjects**	**Areas used to decipher single letters and isolated words**			**Areas used to read paragraphed text**			**Ocular movements to entirely uncover presented words**		
	**Before SLO training**	**Immediately after SLO training**	**Three months after SLO training**	**Before SLO training**	**Immediately after SLO training**	**Three months after SLO training**	**Before SLO training**	**Immediately after SLO training**	**Three months after SLO training**

**MT**	PRL: 1	PRL: 1	PRL: 1	PRL: 1	PRL: 1 nPRLs: 1	PRL: 1 nPRLs: 2	No	Yes	No
**SL**	PRL: 1	PRL: 1 TRL: 1	PRL: 1 TRL: 1 nPRLs: 2	PRL: 1	PRL: 1 nPRLs: 2	PRL: 1 TRL: 1 nPRLs: 2	No	Yes	Yes
**LG**	PRL: 1	PRL: 1 TRL: 1 nPRL: 1	PRL: 1 TRL: 1 nPRL: 1	PRL: 1	PRL: 1 TRL: 1 nPRLs: 1	PRL: 1 TRL: 1	No	Yes	Yes
**AA**	PRL: 1	PRL: 1 TRL: 1 nPRL: 1	PRL: 1 TRL: 1 nPRL: 1	PRL: 1	PRL: 1 TRL: 1	PRL: 1 TRL: 1	Yes	Yes	Yes
**GM**	PRL: 0	TRL: 1	TRL: 1 nPRLs: 2	PRL: 0	TRL: 1	TRL: 1	Yes	Yes	Yes

PRL: initial PRL; TRL: examiner's selected TRL; nPRL: additional newly self-selected PRLs. Following the training four subjects (SL, LG, AA, GM) used either the examiner's selected TRL and/or newly self-selected PRLs both for isolated words' deciphering and for paragraphed text reading. Note that, before training, only two subjects were able to perform eye movements to entirely uncover isolated words, either sequentially using the initial PRL, or at once using the examiner's selected TRL. Immediately following training, all subjects were able to achieve the task. In four out of five subjects this capacity was retained three months following SLO training. Note also that the subject that did not retain the ability to uncover words also did not retain any functional training gains.

All subjects gave their written informed consent for the procedure and testing methods, all of which were in accordance with the Declaration of Helsinki and the study had been accepted by the ethical committee for human experimentation of Geneva University Hospitals.

### Reading strategies for isolated words and paragraphed text

#### Initial evaluation

The results of the initial evaluation step showed that for reading isolated words and paragraphed text four (MT, SL, LG, AA) out of five subjects had spontaneously developed an initial PRL that fulfilled the needs for detailed discrimination capacity although it prevented global viewing. Indeed, the PRL showed better visual acuity than the examiner's selected TRL, but was not optimally located to allow the viewing of the whole word, thus altering reading ability (figure 3A, 4, 5A).

Three subjects (MT, SL, LG) had an initial PRL to the left of the scotoma and consequently ignored the end of the words (figure 3A, 5A and 6A). Two of these subjects (SL, LG) were additionally unable to perform search movements to detect single letters and isolated words initially projected within the scotoma, and to relocate the projected image onto a healthy peripheral area. The third subject (MT) showed an additional small scotoma in the vicinity of the left border of the central scotoma on the SLO picture that repeatedly superimposed on parts of the scrutinized characters (figure 1, 5). We trained these three subjects (MT, SL, LG) to use an examiner's selected TRL above the scotoma on the SLO image (i.e. below the scotoma in the visual field).

The fourth subject (AA) had an initial PRL located above the scotoma on the SLO image, but too close to the scotoma's border, the upper part of the letters scrutinized being often projected into the scotoma. Consequently he often missed the center of presented words. We trained him to use an examiner's selected TRL located on the same side of the scotoma as the initial PRL, i.e. above the lesion on the SLO image, but farther from the scotoma's border (figure 1).

The remaining fifth subject (GM) had not developed a PRL despite previous rehabilitation. He was found to use an ill-defined area with preserved global viewing but non-optimal spatial resolution. In addition, he presented a highly instable fixation.

Based on the results of visumetry, he was trained to use an area below the scotoma on the SLO picture, which presented a better acuity than the initial PRL while still preserving global viewing capacity (figure 1).

#### Post-training evaluations

Immediately after SLO training, analyses of reading strategies revealed that four subjects (SL, LG, AA, GM) used the examiner's selected TRL, or additional newly self-selected PRLs (figure 3B and 6B) alone or in combination with their initial PRL for reading isolated words and paragraphed text. One of these subjects (GM) (no defined PRL before SLO training) used the examiner's selected TRL alone. The remaining fifth subject (MT) showed an improved ability to scan the word but only used the initial PRL (figure 5B).

Three months following SLO training, four subjects (SL, LG, AA, GM) consistently used the examiner's selected TRL either in isolation (GM), or in combination with the initial PRL (LG, AA, SL) for reading both isolated words and paragraphed texts (table 3, figures 4 and 7). The use of newly developed self-selected PRLs was observed in three subjects (SL, AA, GM) to read isolated words and in two subjects to read paragraphed text (MT, SL). Interestingly, one subject (MT) lost the ability to perform eye movements to scan the entire words, but kept some adaptation, i.e. used newly self-selected PRLs, during text reading (table 2).

### ETDRS visual acuity

**Figure 8:**
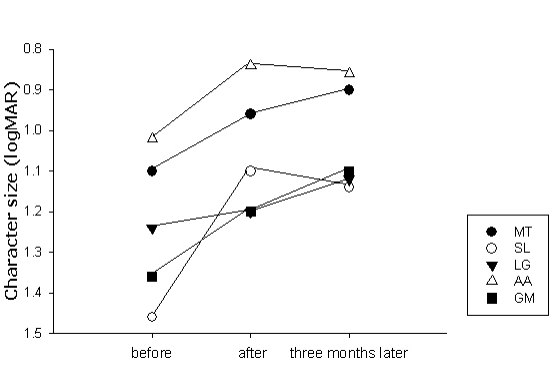
**Evolution of ETDRS visual acuity**. Measures are reported for each subject. *before: *before SLO training; *after: *immediately after SLO training; *three months later: *three months following SLO training. Changes in ETDRS values could be considered significant when greater than 0.2 logMAR [38, 39]. Note that ETDRS visual acuity consistently increased with the SLO training and, globally, gains were retained three months later.

ETDRS values are reported in table 2 and figure 8. Visual acuity evolution across the three evaluation periods was analyzed using a one-way repeated measure ANOVA. This analysis yielded a significant difference (F(2,3) = 17.85; p = 0.022). Values measured before and three months following the SLO training significantly improved compared to those measured before the training process (Bonferroni pairwise comparison p = 0.012), while the difference was marginally significant between acuities measured before and immediately after SLO training (Bonferroni pairwise comparison p = 0.085). However when considering individual data, the majority of improvements were no greater than the test repeatability of 0.2 [34,35].

### Threshold character size for single letters and isolated words

**Figure 9:**
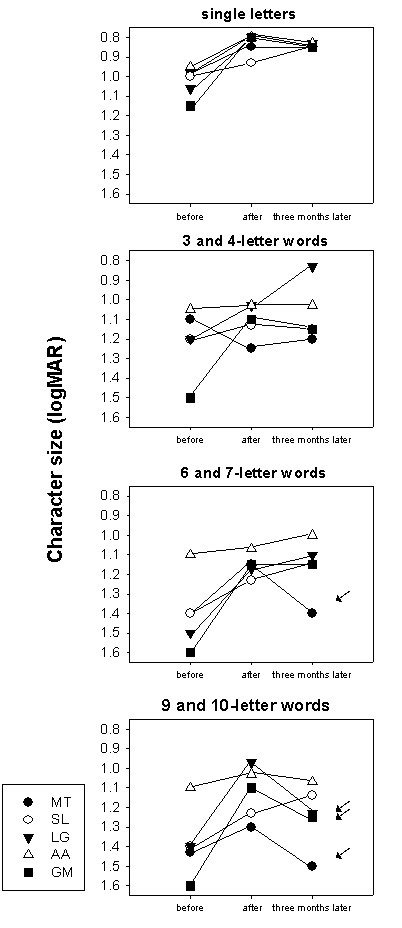
**Evolution of threshold character size for single letters and isolated words**. Threshold character sizes' values in logMAR, are provided for each subject. *before: *before SLO training; *after: *immediately after SLO training; *three months later: *three months following SLO training. Isolated letters and word length categories are considered independently. With subject GM, threshold character size for 6 and 7 and 9 and 10 letter words could not be determined before training, because he was unable to decipher the largest presented stimuli (1.5 logMAR). Therefore, in the graph, he was assigned the minimum 1.6 logMAR level. Note the invariance of the results for isolated letters, contrasting with the variability of the changes obtained for isolated words. Loss of training gains mainly occurred when deciphering longer words. In this regard, measures in subject LG are particularly illustrative, showing continuous improvement in reading single letters and 3/4-letter words, stabilized performance for 6 and 7-letters words, results which contrast with the gain loss observed for 9 and 10-letter words.

Threshold character sizes for single letters and isolated words are shown in table 2 and figure 9. A three-way repeated measure ANOVA with subjects (MT, SL, LG, AA, GM), stimulus types (single letters, 3 and 4 letter-words, 6 and 7 letter-words and 9 and 10 letter-words) and evaluation periods (before, at the end of training and three months later) was conducted to explore the impact of the training procedure as measured by the threshold character size.

There was a highly significant main effect of the evaluation periods (F(2, 19) = 46.04; p < 0.0005). The post-hoc analysis using Bonferoni correction showed that all subjects benefited from the training procedure both, immediately after SLO training (mean ± standard error: before = 1.255 ± 0.017, immediately after SLO training = 1.055 ± 0.016; p < 0.0005) and three months later (1.084 ± 0.014; p < 0.0005). There was, however, no significant evolution after completion of the training procedure (p = 0.563).

There was also a main effect of stimulus type confirming that threshold character size varied as a function of word length (F(3, 20) = 73.921; p < 0.0005). Threshold character size for single letters (0.9 ± 0.019) was lower than for any other word lengths and threshold for 3 and 4 letter-words (1.13 ± 0.019) was lower than those for 6 and 7 (1.23 ± 0.019) and 9 and 10 letter-words (1.26 ± 0.019; Newman-Keuls: p < 0.05). Additionally, the retention of the training gains varied with stimulus types (interaction evaluation periods and stimulus type: F(6, 38) = 3.135; p = 0.014). To further investigate the retention of the training gains on the different stimulus types we conducted a post-hoc analysis using Bonferroni adjustment for multiple comparisons with α = 0.00125. Subjects read significantly smaller character sizes immediately after the end of the SLO training procedure at all word lengths but 3/4 letter-words, despite the fact that a slight improvement was noticeable for the latter (1 letter: before = 1.02 ± 0.029, immediately after SLO training = 0.82 ± 0.022; p = 0.006; 6 and 7-letter words: before = 1.41 ± 0.062, immediately after SLO training = 1.15 ± 0.027; p = 0.008; 9 and 10-letter words: before = 1.38 ± 0.057, immediately after SLO training = 1.13 ± 0.045; p = 0.006; 3 and 4-letter words: before = 1.21 ± 0.062, immediately after SLO training = 1.10 ± 0.045; p = 0.513). Three months following the training procedure, gains were retained for single letters and 6 and 7-letter words, as shown by the non-significant difference between results obtained at the end of training and three months later (1 letter: three months later = 0.86 ± 0.016; p = 1.0; 6 and 7 letter-words: three months later = 1.13 ± 0.056; p = 1.0). In contrast, performances were not retained for 9 and 10-letter words (three months later = 1.28 ± 0.057; p = 0.02) and returned to the initial score (p = 0.32). For 3 and 4-letter words the statistical test did not reveal any additional change over the whole procedure either (immediately after SLO training – three months later: p = 1.0: before – three months later: p = 0.456).

Another main result was that subjects benefited differently from the training procedure, as shown by the interaction between evaluation periods and subjects (F(8, 38) = 5.371; p < 0.0005). Immediately after the SLO training procedure, all subjects had improved their performances regardless of the type of stimulus. Three months later subjects SL, LG and AA totally retained and even slightly improved, training gains. Subject GM did not fully preserve the improvements, even if his performances were far better than before the SLO training. Subject MT did not retain training gains at all (figure 9). Despite the fact that the interaction subjects, stimulus type and evaluation periods was not significant (F(24; 38) = 0.1014; p = 0.497), it is interesting to describe individual behavior. When looking at individual data (figure 9), it appears that all subjects had better performances three months following the training procedure than before for 3 and 4-letter words. For 6 and 7-letter words, three subjects (SL, LG, AA) progressed in deciphering capacities, one subject (GM) retained training benefits, and the remaining subject (MT) partially lost training gains. For the 9 and 10-letter words, one subject (SL) improved, three subjects (LG, AA, GM) partially lost the gains, and the remaining subject (MT) lost all benefits of the SLO training (see arrows in figure 9). Finally there was a main effect of subjects, as expected regarding the individual variations of threshold character sizes (F(4, 20) = 16.443; p < 0.0005). The interaction subject and stimulus type was not significant (F(12; 20) = 1.100; p = 0.087).

### Paragraphed text reading

**Figure 10:**
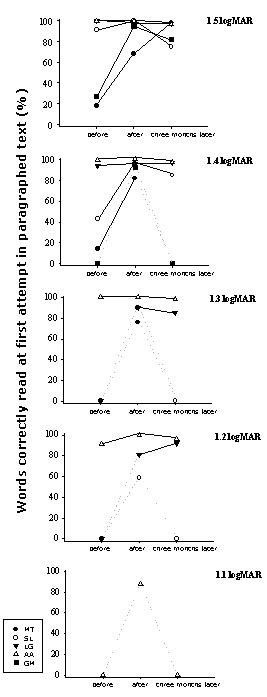
**Evolution of paragraphed text reading performance over the training process**. This figure shows the percentage of words correctly read at the first attempt in paragraphed text before, immediately after, and three months following SLO training. Percentage is plotted for each character size – from the largest size presented (1.5 logMAR, upper graph) to the smallest readable character size (1.1 logMAR, lower graph). The percentage was assigned to zero when, after attempting to read, the subject felt unable to decipher a single word. Dashed are used when a value was assigned to zero at one of the training phases. It appears that, at the end of the training procedure, all subjects were able to read correctly about 82% or more words at first attempt at the largest character size presented. Note that, previous to SLO training, two of these subjects had demonstrated a very low percentage at that character size. The three subjects (SL, LG, AA) who, already before training, exhibited a high percentage of words correctly read, improved their ability to read smaller character sizes immediately following SLO training. Finally, it should be emphasized that gains observed at the end of training period were often not retained three months later.

Threshold character size for paragraphed text and percentage of words read are shown in table 2 and figure 10. Immediately following SLO training, three subjects (SL, AA, GM) became able to read paragraphed text one character size smaller (0.1 logMAR) and one subject (LG) could read two character sizes smaller (0.2 logMAR) than evaluated before training (table 2). In all subjects, the proportion of words correctly read at the first attempt, i.e. reading accuracy, increased with training (figure 9). Three months following the completion of the SLO training procedure, the smallest readable character size for paragraphed text was similar to that of the initial evaluation in four subjects (MT, SL, AA, GM), but the percentage of words correctly read had increased (figure 9) allowing subjects to have an adequate comprehension on smaller character sizes considering that an accuracy of 85% allows a complete understanding of the text (27).

### Patients' comments on the modification of their visual perception before and after the training

We asked the patients to give a subjective evaluation of the influence of the SLO training on their everyday life. All patients reported less visual fatigue and improved awareness of the location of the scotoma. MT reported that two weeks after the completion of the SLO training, she had the impression of an improvement in reading. SL noticed that SLO training enabled a more efficient reading of the panels in the street as well as bus numbers. LG could not report specific subjective improvements but said that, in general, she better coped with her visual field defect. AA applied the taught strategy to other visual tasks. She said she recovered the ability to hand-knit. After the SLO training process, GM started to use a closed circuit television (CCTV) and was able to read with it. He stated that the SLO training allowed him to locate his scotoma in his visual field and, as a result, several visual tasks were easier to accomplish.

## Discussion

SLO training in subjects with macular conditions had been emphasized previously [21]. This technique allows local visual acuity measurements, estimation of potential global viewing capacity in different areas of the retina and direct understanding of the nature of reading problems [21,36]. It also enables the guidance of subject's fixation onto the presented words with a continuous interaction between the subject and the examiner during the training session. The particularity of our training procedure consisted of an attempt to structure new oculomotor behavior involving the use of additional fixation points, without preventing the patients to use his initially spontaneously developed PRL. We also established the schedule of our training process based on recent findings in neurorehabilitation showing that intensive and short-term training was beneficial [reviewed in 25], and applied, for example, in individuals with chronic aphasia [24]. Thus, as retaining new oculomotor behaviors require brain plasticity and as the occurrence of such phenomenon is comparable with those occurring in patients suffering from other neural damages, we schedule the SLO training accordingly.

Based on our first results, we can already discuss possible events underlying the adaptation to a second fixation area.

### Immediate functional benefits and gains retention

Our analyses of reading strategies demonstrated essentially that our SLO training allowed subjects with limited global viewing to acquire the capacity to perform eye movements, which allow the perception of the presented words in their whole. The newly adapted oculomotor behavior included the use of the examiner's selected TRL, either in isolation or in combination with the initial spontaneously developed PRL and the associated use of newly self-selected PRLs. Three subjects used the examiner's selected TRL in combination with other areas, whereas the remaining subject, who had no defined initial PRL, used only the examiner's selected. Interestingly, the development of the newly self-selected PRLs and new oculomotor patterns suggest that the structured training procedure, aiming primarily at developing an examiner's selected TRL, in fact is likely to initiate a more global functional adaptation process. As already suggested in neuro-rehabilitation studies, training might trigger general changes in adaptation strategies and thus may "prime" the brain for future learning [26].

In our subjects, the adaptation of reading strategies was associated with improvements measured in different reading related tasks. The only subject (MT) who showed no adaptation of oculomotor behavior also did not improve performances in reading related tasks. ETDRS acuity and threshold character size for single letter measurements evolved, as expected, similarly throughout the training. Increased visual acuity was likely related to an increased ability to detect and then relocate stimuli that fell into scotoma's area. One might also consider whether gains in ETDRS acuity and threshold character size for single letters and isolated words observed following SLO training were non-specific results of the training process, and unrelated to the use of the SLO. This hypothesis however, is unlikely, as all subjects had previously undergone a conventional low vision training procedure, which proved to be unsuccessful in allowing isolated words to be deciphered.

Assessment conducted three months following the end of the SLO training showed that gains persisted and that improvements remained for reading single letters but were not retained for 9 and 10-letter words. Deciphering 9 and 10-letter words requires both accurate fixation and adapted saccadic movements, probably more so than those required for isolated letters and short words, which necessitate essentially accurate eccentric fixation. Differences between various reading tasks in terms of gain retention presumably reflect the variation in complexity of mechanisms involved in specific adaptation processes. Indeed, it is conceivable that complex tasks more demanding in terms of plasticity may be more difficult to retain and/or need more extensive training. A similar variability in the retention of performances as a function of the complexity of the task was observed in 400 patients who had followed short-term, intensive procedures for rehabilitation on motor tasks [37].

### Eye movements' adaptation

The adaptation of the oculomotor system studied here involved at least two different mechanisms: the first one to achieve eccentric fixation, and the second to reorganize the control of saccades around the coordinates of the initial PRL and the examiner's selected TRL. The distinction between these mechanisms and their respective complexity has been demonstrated experimentally. Heinen and Skavenski (1992) [38] found in adult monkeys that one day after bilateral foveal lesions, fixation was performed with a new retinal locus, although saccades at this stage of adaptation still maladaptively brought visual targets onto the lesioned fovea. Subsequently, over a period of several weeks, saccades trajectories progressively changed and targets projected directly onto the intact retina [38]. In our study, individual practice of oculomotor strategies during the period following the SLO training might also have influenced the degree of retention of reading ability. Particularly, in one subject (MT) who could not pursue any reading practice at home, the gains observed immediately after SLO training were eventually reduced, and even return to baseline performances. We therefore propose that to retain improvements on complex tasks "brush up" periods of training might be advised following structured procedure.

It is of interest to investigate to what extent the improvements obtained following training for isolated words might reflect subjects' increased ability to read paragraphed texts in everyday' life conditions. We previously reported in subjects with macular scotoma, a strong correlation between the threshold character size for reading words longer than five letters and for reading paragraphed text in the SLO [39]. Consequently, following reading training, improvements on longer words are likely to be associated with an improvement on paragraphed text, although the latter requires a more complex adaptation of eye movements – to perform accurate return sweeps, for example. In this pilot training study we showed that patients did not acquired the capacity to read paragraphed text of smaller character sizes but improved the number of words correctly read at first attempt and thus reached levels of complete text comprehension. We limited our analysis of paragraphed text reading to an evaluation of reading threshold character size and the determination of the percentage of words correctly read at first attempt because the SLO is not adapted to determine reading speed as a important number of images are lost while the subject performs eye movements.

## Conclusion

In subjects with macular disorders, we previously suggested that learning to control eye movements improved the use of the PRL and facilitates the development of additional PRLs [20]. Here we proposed a procedure allowing the training of the use of multiple PRLs in reading related tasks, which seemed to favor the development of additional individual reading strategies involving the examiner's selected TRLs and newly self-selected PRLs to bypass problems linked to the presence of the central macular lesion. SLO training aiming at combining the use of multiple PRLs is feasible and likely to improve reading ability. The SLO has unfortunately been out of production for many years and for this reason is presently not a widespread instrument. However, our study suggested that similar and simpler devices allowing real time view of the retina and projection of chosen stimuli might be introduced into the market for the purpose of reading training, particularly as the incidence of AMD is expected to improve in the following years. The first results with such a training that we obtained on five patients are encouraging but to reach definite results the training of a larger group of subjects and a comparison of data with those of a control group would be more compelling.

## Competing interests

The author(s) declare that they have no competing interests.

## Authors' contributions

AD participated in the design of the study, the acquisition, analysis and interpretation of the data, and the drafting of the final manuscript. MG participated in the design of the study, the interpretation of the data and the drafting if the final manuscript. ARW participated in the design of the study and the drafting of the manuscript. CM designed the computer software necessary to all analyses of the data. ENL performed the statistical analysis and revised the manuscript. AS participated in the design of the study and in the drafting of the final manuscript. ABS participated in the design of the study, in the interpretation of data and in drafting the final manuscript. All authors read and approved the final manuscript.

## Pre-publication history

The pre-publication history for this paper can be accessed here:


